# Multiple serum biomarkers for predicting suicidal behaviours in depressive patients receiving pharmacotherapy

**DOI:** 10.1017/S0033291722001180

**Published:** 2023-07

**Authors:** Jae-Min Kim, Hee-Ju Kang, Ju-Wan Kim, Wonsuk Choi, Ju-Yeon Lee, Sung-Wan Kim, Il-Seon Shin, Min-Gon Kim, Byung Jo Chun, Robert Stewart

**Affiliations:** 1 Department of Psychiatry, Chonnam National University Medical School, Gwangju, Korea; 2 Department of Internal Medicine, Chonnam National University Hwasun Hospital, Chonnam National University Medical School, Hwasun, Korea; 3 Department of Chemistry, School of Physics and Chemistry, Gwangju Institute of Science and Technology, Gwangju, South Korea; 4 Department of Emergency Medicine, Chonnam National University Medical School, Gwangju, Republic of Korea; 5 King's College London (Institute of Psychiatry, Psychology and Neuroscience), London, UK; 6 South London and Maudsley NHS Foundation Trust, London, UK

**Keywords:** Depression, multimodal biomarker, pharmacotherapy, prediction, suicidality

## Abstract

**Background:**

Predictive values of multiple serum biomarkers for suicidal behaviours (SBs) have rarely been tested. This study sought to evaluate and develop a panel of multiple serum biomarkers for predicting SBs in outpatients receiving a 12-month pharmacotherapy programme for depressive disorders.

**Methods:**

At baseline, 14 serum biomarkers and socio-demographic/clinical characteristics including previous suicidal attempt and present suicidal severity were evaluated in 1094 patients with depressive disorders without a bipolar diagnosis. Of these, 884 were followed for increased suicidal severity and fatal/non-fatal suicide attempt outcomes over a 12-month treatment period. Individual and combined effects of serum biomarkers on these two prospective SBs were estimated using logistic regression analysis after adjustment for relevant covariates.

**Results:**

Increased suicidal severity and fatal/non-fatal suicide attempt during the 12-month pharmacotherapy were present in 155 (17.5%) and 38 (4.3%) participants, respectively. Combined cortisol, total cholesterol, and folate serum biomarkers predicted fatal/non-fatal suicide attempt, and these with interleukin-1 beta and homocysteine additionally predicted increased suicidal severity, with clear gradients robust to adjustment (*p* values < 0.001).

**Conclusions:**

Application of multiple serum biomarkers could considerably improve the predictability of SBs during the outpatient treatment of depressive disorders, potentially highlighting the need for more frequent monitoring and risk appraisal.

## Background

Suicide is a major cause of death globally, with approximately 800 000 people dying by suicide every year (Naghavi & Global Burden of Disease Self-Harm Collaborators, 2019), and suicidal ideation and attempt are 10–20 times more common (Mann, 2003). A rational first step for monitoring and preventing suicidal behaviour (SB) is the identification of risk factors, which is not easy because it relies on subjective reports (Blasco-Fontecilla, Lopez-Castroman, Giner, Baca-Garcia, & Oquendo, 2013). Since suicide has distinctive pathophysiology based on stress-diathesis models (Oquendo et al., 2014), the application of objective biological tests could improve the predictability of SB (Sudol & Mann, 2017).

Peripheral blood biomarkers have several advantages in this respect, given their accessibility, cost-effectiveness, and ease of collection even in those with significant suicidality. A variety of peripheral blood biomarkers relevant to the pathophysiology of SB have been evaluated. Markers related to the hypothalamic-pituitary-adrenal (HPA) axis, a major stress-response system, have received particular attention; cortisol is an effector hormone for the HPA axis, but its relations with SB have been inconsistent (O'Connor, Ferguson, Green, O'Carroll, & O'Connor, 2016). Additionally, the serotonergic system is involved in both stress and diathesis of SB (Mann, 2013), and low blood serotonin levels have been found to be related to SB (Tyano et al., 2006). Markers of immune and inflammatory function have long been investigated, given their connection to the HPA axis and serotonin system, with markers investigated including high-sensitivity C-reactive protein (hsCRP), pro-inflammatory cytokines such as tumour necrosis factor-alpha (TNF-*α*), interleukin-1 beta (IL-1*β*), and IL-6, and anti-inflammatory cytokines such as IL-4 and IL-10 (Black & Miller, 2015; Choi et al., 2021). Lipids may also play a role in the pathophysiology of SB, given that low cholesterol levels may impair central serotonin transportation (Engelberg, 1992; Wu et al., 2016), and leptin and ghrelin, which may affect lipid concentrations, have also been investigated (Atmaca et al., 2006; González-Castro et al., 2021). Nutrients that could protect against cellular damage by stresses and affective disorders are potential biomarker candidates including folate, omega 3 fatty acids, and homocysteine (Du et al., 2016). The neuroplastic function is important in the adaptation of CNS to external stresses, and brain-derived neurotrophic factor (BDNF) has been the most frequently studied marker of this (Eisen et al., 2015).

Despite extensive previous research, the blood biomarkers suggested are rarely used in clinical practice for a number of reasons. To begin with, diagnostic and screening values of individual biomarkers have been disappointing in gauging the risk of SB (Blasco-Fontecilla & Oquendo, 2016). Combinations of two unrelated biomarkers might provide better results than a single marker, but the accuracy of this approach was still unsatisfactory (Coryell & Schlesser, 2007; Jokinen, Martensson, Nordstrom, & Nordstrom, 2008). Multiple biomarkers covering various functional systems in combination might further increase the accuracy (Sudol & Mann, 2017), but this approach has not been taken to date. In addition, most studies have evaluated SB in terms of a previous suicidal attempt or present suicidal severity using case–control study designs rather than as a prospective outcome (Black & Miller, 2015; Eisen et al., 2015; O'Connor et al., 2016; Wu et al., 2016).

The suicide mortality rate in Korea was 24.6 per 100 000 in 2019, the highest for countries in the Organization for Economic Cooperation and Development (OECD) (OECD, 2021). SB is strongly associated with depressive disorder (Dong et al., 2019). Using data from a prospective study of Korean patients with depressive disorders receiving a 12-month stepwise pharmacotherapy, we aimed to develop and evaluate multiple serum biomarkers panel covering distinctive functional systems for predicting SB.

## Methods

### Study outline

This study was a secondary analysis as a component of the MAKE Biomarker discovery for Enhancing anTidepressant Treatment Effect and Response (MAKE BETTER) programme. Details of the parent study have been published as a design paper (Kang et al., 2018) and the study was registered with cris.nih.go.kr (identifier: KCT0001332). All data on socio-demographic and clinical characteristics at baseline, and treatment-related variables at follow-up examinations during the acute treatment phase (evaluated at 3, 6, 9, 12 weeks) and during the continuation treatment phases (evaluated at 6, 9, and 12 months) were obtained using a structured clinical report form (CRF) by clinical research coordinators who were blind to treatment modalities. These staffs were trained in CRF implementation and data collection methods by the research psychiatrists. Patients' data were recorded on a CRF, registered on the website of the MAKE BETTER study (http://icreat.nih.go.kr/icreat/webapps/com/hismainweb/jsp/cdc_n2.live) within 3 days, and monitored by data management centre personnel. This study was approved by the Chonnam National University Hospital Institutional Review Board (CNUH 2012-014).

### Participants

Patients with depressive disorders were consecutively recruited from March 2012 to April 2017 from those who had visited the outpatient psychiatric department of Chonnam National University Hospital. Inclusion criteria were: (i) aged older than 7 years; (ii) diagnosed with MDD, dysthymic disorder, or depressive disorder not otherwise specified (NOS), using the Mini-International Neuropsychiatric Interview (MINI) (Sheehan et al., 1998), a diagnostic psychiatric interview applying Diagnostic and Statistical Manual of Mental Disorders, Fourth Edition (DSM-IV) criteria (American Psychiatric Association, 1994); (iii) Hamilton Depression Rating Scale (HAMD) (Hamilton, 1960) score ⩾ 14; (iv) able to complete questionnaires, understand the objective of the study, and sign the informed consent form. Exclusion criteria were: (i) an unstable or uncontrolled medical condition; (ii) unable to complete the psychiatric assessment or comply with the medication regimen, due to a severe physical illness; (iii) current or lifetime DSM-IV diagnosis of bipolar disorder, schizophrenia, schizoaffective disorder, schizophreniform disorder, psychotic disorder NOS, or other psychotic disorder; (iv) history of organic psychosis, dementia, epilepsy, or seizure disorder; (v) history of anticonvulsant treatment; (vi) hospitalisation for any psychiatric diagnosis apart from depressive disorder (e.g. alcohol/drug dependence); (vii) electroconvulsive therapy received for the current depressive episode; (viii) pregnant or breastfeeding. All inclusion instances represented new treatment episodes – i.e. taking newly initiated antidepressant treatment – whether depressive symptoms were first-onset or recurrent. All participants reviewed the consent form and written informed consent was obtained. To reflect real-world settings for all depressive outpatients, participants were enrolled irrespective of age. For participants aged under 16, written consent was obtained from a parent or legal guardian, and written assent was obtained from the participant.

### Baseline measures

#### Serum biomarkers

Participants were instructed to fast from the night before for morning blood sampling, and to sit for 25–45 min quietly and relax before blood samples were acquired. Serum samples were separated and immediately frozen at −80 °C at the clinical laboratories of the CNUH. All laboratory measurements were conducted by the Global Clinical Central Lab (Yongin, Korea) blind to patients' status. Fourteen blood biomarkers representing six functional systems were pre-selected based upon existing evidence (Lee & Kim, 2011; Sudol & Mann, 2017), and there were no others that measured but not presented here. These were measured using the following methods:

- HPA axis
  - cortisol: Cobas Cortisol II electrochemiluminescence Immunoassay (Roche, Vilvoorde, Belgium).
- Serotonergic
  - serotonin: ClinRep high-performance liquid chromatography kit (Recipe, Munich, Germany).
- Immune and inflammatory
  - hsCRP: Tina-quant C-reactive protein (latex) high sensitive assay (Roche, Vilvoorde, Belgium).
  - TNF-*α*: Quantikine^®^ HS ELISA Human TNF-*α* Immunoassay (R&D Systems, Minneapolis, USA).
  - IL-1*β*, IL-6, IL-4, and IL-10: Human High Sensitivity T Cell Magnetic Bead Panel (EMD Millipore, Billerica, USA).
- Lipids
  - total cholesterol: L-type CHO M cholesterol oxidase method kit (Wako Pure Chemical Industries, Osaka, Japan).
  - leptin: Human Leptin ELISA (BioVendor Laboratory Medicine, Inc., Modrice, Czech Republic).
  - ghrelin: GHRELIN (Total) radioimmunoassay kit (EMD Millipore, Billerica, USA).
- Nutritional
  - folate: Cobas Elecsys Folate III electrochemiluminescence Immunoassay (Roche, Vilvoorde, Belgium).
  - homocysteine: ARCHITECT Homocysteine 1L71 Kit (Abbot, Wiesbaden, Germany).
- Neuroplastic
  - BDNF: Quantikine^®^ ELISA Human BDNF Immunoassay (R&D Systems Inc., Minneapolis, USA).

#### Suicidal behaviours

The previous suicidal attempt was defined by using the ‘suicide attempt’ category of the Columbia Classification Algorithm of Suicide Assessment (C-CASA) (Posner, Oquendo, Gould, Stanley, & Davies, 2007). Participants were asked whether they had potentially self-injurious behaviour, associated with at least some intent to die, as a result of the act before the baseline evaluation. Equivocal intention to die at that time of an intentional self-harm act also defined as a suicide attempt. Positive responses to these were defined to have previous suicidal attempt. However, self-injurious behaviours with no suicidal intention or unknown intention were not included from the definition. Baseline suicidal severity was evaluated by the observer-rated Brief Psychiatric Rating Scale (BPRS) (Overall & Gorham, 1962) suicidality item score. Participants were asked ‘Have you felt that life wasn't worth living? Have you thought about harming or killing yourself? Have you felt tired of living or as though you would be better off dead? Have you ever felt like ending it all?’ If participants reported suicidal ideation, further questions were asked ‘How often have you thought about this? Do you have a specific plan?’. Participants' self-report was recorded as a Likert scale (1 = not present, 2 = very mild, 3 = mild, 4 = moderate, 5 = moderately severe, 6 = severe, 7 = extremely severe). Previous studies used different cut-offs ranging from 2 to 5 for capturing suicidal severity (Fedyszyn, Robinson, Matyas, Harris, & Paxton, 2010; Tor, Abdin, Hadzi-Pavlovic, & Loo, 2020) without any standardised criterion. For the present study, this was divided into lower [score 1~3] *v.* higher [score 4~7] suicidal severity groups arbitrarily for capturing moderate to severe suicidal severity.

#### Covariates

Data on the following socio-demographic characteristics were obtained: age, sex, year of education, marital status (currently married or not), cohabitation status (living alone or not), religion (religious observance or not), occupational status (current employed or not), and monthly income level (above or below 2000 USD). Clinical characteristics assessed comprised diagnoses of depressive disorders (MDD or other depressive disorders) with the following specifiers: melancholic or atypical features, onset age and illness duration, number of previous depressive episodes, duration of the present episode; in addition, family history of depression, number of concurrent physical disorders (applying a questionnaire asking for 15 systems or diseases), and smoking status (current smoking or not) were ascertained. Depressive and anxiety symptoms were evaluated by the Hospital Anxiety Depression Scale depression (HADS-D) and anxiety (HADS-A) subscales, respectively (Zigmond & Snaith, 1983), and alcohol-related problems by the Alcohol Use Disorders Identification Test (AUDIT) (Saunders, Aasland, Babor, de la Fuente, & Grant, 1993). Higher scores indicate more severe symptomatology.

### Stepwise pharmacotherapy

The treatment steps and strategies have been previously published (Kim et al., 2020) and in general sought to achieve an approach that was both standardised and naturalistic. Before the treatment commencement, a comprehensive examination was carried out of clinical manifestations, illness severity, physical comorbidities and medication lists, and history of prior treatments. In the first step, patients received antidepressant medication, considering these patient characteristics and existing treatment guidelines (Bauer et al., 2013; Kennedy et al., 2016; Malhi et al., 2015) for 3 weeks. General effectiveness and tolerability were evaluated for going ahead with next-step measurement-based treatments (Guo et al., 2015). In cases of inadequate improvement or intolerable adverse events, patients were directed to choose whether they would prefer to stay in the present step or get in the next step treatment by switching antidepressants (S), augmenting with other drugs (A), the combination of other antidepressants (C), S + A, S + C, A + C, and S + A + C strategies. In settling on treatment strategies, patients' preferences were given priority in order to maximise adherence and treatment outcomes (Swift & Callahan, 2009).

### Prospective suicidal behaviours

For assessing ‘increased suicidal severity’, the BPRS suicidality item score was re-evaluated during the 12-month pharmacotherapy period at 3, 6, 9, and 12 weeks, and thereafter at 6, 9, and 12 months post-baseline. Any instance of an increase in the score evaluated the follow-up points compared to the baseline score was defined as increased suicidal severity. Fatal/non-fatal suicide attempt included suicidal attempt defined as above and death by suicide during the 12-month pharmacotherapy period.

### Statistical analysis

Baseline socio-demographic and clinical characteristics including assessment scales, and treatment step during the 12-month pharmacotherapy period were compared by the presence of previous suicidal attempt and by lower *v.* higher baseline suicidal severity groups using *t* tests or χ^2^ tests as appropriate. Covariates for further adjusted analyses were selected from those characteristics associated at conventional levels of statistical significance (*p* < 0.05) in these analyses, having considered collinearity between variables. For estimating individual associations with prospective SBs, baseline serum biomarker levels were compared by the increased suicidal severity and by fatal/non-fatal suicide attempt during the 12-month pharmacotherapy using Mann–Whitney *U* tests. For those biomarkers showing statistical significance (*p* < 0.05), optimal cut-offs with sensitivities and specificities were calculated against the two SBs by using area under receiver operating curve (AUROC) analyses. Odds ratios and 95% confidence intervals (ORs and 95% CIs) for the two SBs were estimated by the dichotomised (as favourable *v.* unfavourable group) optimal cut-offs of each biomarkers using logistic regression analysis after adjustment for relevant covariates. Bonferroni correction was used to maintain an overall type 1 error rate of 0.05 for the individual associations between individual biomarkers and suicidal behaviours.

The effects of multiple biomarkers on prospective SBs were evaluated in two ways. First, summed up scores were calculated from the significant biomarkers, and then associations between the increased number of biomarkers and SBs were investigated using logistic regression analysis after adjustment for covariates. ORs and 95% CIs were calculated for each group with the 0 score as a reference. Second, a continuous multi-biomarker score was estimated using the following equation based on the significant biomarkers: *H* = (*β*1 × biomarker A) + (*β*2 × biomarker B), and so on, where *β*1 and *β*2 denote the estimates of beta coefficients for biomarkers A and B, and were obtained by fitting the logistic regression model for each SB. These kinds of analytic methods on summations of the number of increased biomarkers and on weighted continuous multi-biomarker scores were frequently used in longitudinal disease outcome studies (Wang et al., 2006; Zhong et al., 2019). Patients were categorised according to quartiles of the multi-biomarker score. ORs and 95% CIs were calculated for each group with the lowest quartile as reference. Tests for linear trends in ORs were carried out using the increased number of biomarkers and the increased quartiles of a multi-biomarker score.

Additional analyses were carried out to investigate the values of biomarkers for discriminating previous and present SBs by using the same statistical models. All statistical tests were two-sided with a significance level of 0.05. Statistical analyses were carried out using the SPSS 21.0 and STATA 12.0 software.

## Results

### Recruitment

**Fig. 1.**
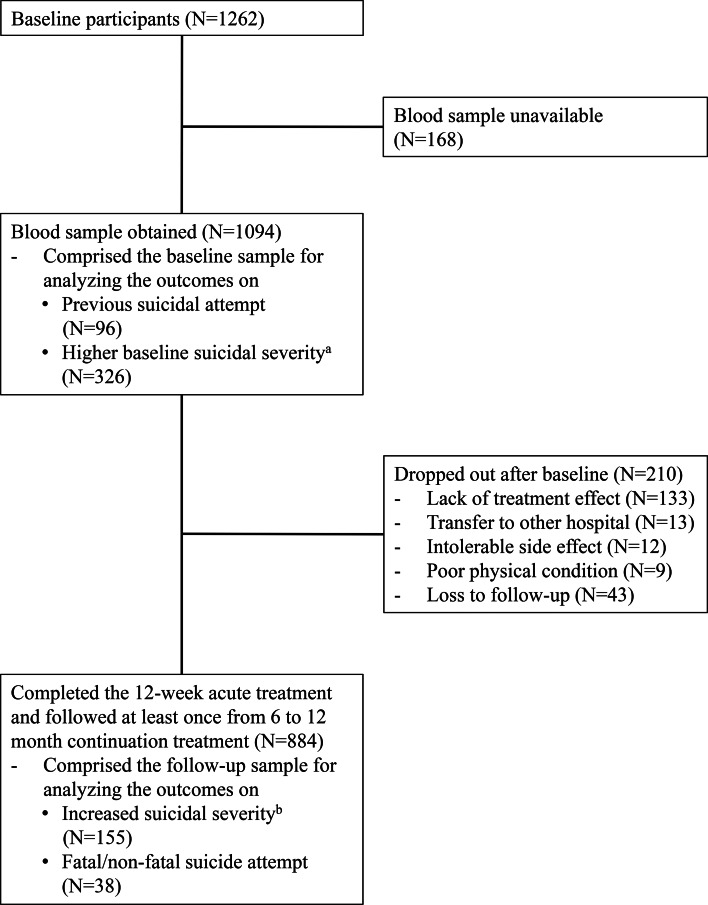
Participant flow. ^a^Brief Psychiatric Rating Scale suicidality item score 4 (moderate) ~ 7 (extremely severe). ^b^Increase in Brief Psychiatric Rating Scale suicidality item score during the follow-up compared to the baseline.

The recruitment process is summarised in Fig. 1. Of 1262 participants evaluated at baseline, 1094 (86.7%) provided a blood sample for measuring serum biomarkers. Of these, 884 (80.8%) completed the 12-week acute treatment and were followed at least once from 6 to 12 months continuation treatment; these comprised the sample for prospective analyses. Descriptive characteristics of the baseline and followed up samples are summarised in online Supplementary Table S1. All participants were aged over 16 (range 17~85). No significant differences in baseline characteristics were found between those with or without a blood sample. However, loss to follow-up at 12 months was significantly associated with unemployed status and melancholic features at baseline.

### Characteristics by previous and present suicidal behaviours

In the baseline sample (*N* = 1094), previous suicide attempt and higher baseline suicidal severity were present in 96 (8.8%) and 362 (33.1%) participants, respectively. Characteristics are compared by these SBs in online Supplementary Table S2 and S3. The previous suicide attempt was significantly associated with younger age, male sex, higher education, unmarried status, no religion, higher monthly income, diagnosis of MDD, atypical depressive features, earlier age at onset, longer duration of illness, higher number of depressive episodes, current smoking status, higher scores on HADS-A and AUDIT, and higher treatment steps over 12-month. A higher baseline suicidal severity was significantly associated with younger age, unmarried status, living alone, no religion, unemployed status, diagnosis of MDD, atypical feature, earlier age at onset, longer duration of illness, a higher number of depressive episodes, current smoking status, higher scores on HADS-D and HADS-A, and higher treatment steps over 12-month. Considering these associations and collinearity between the variables, covariates for further adjusted analyses were selected as follows: age, sex, living alone, religious affiliation, monthly income, atypical feature, number of depressive episodes, number of physical disorders, smoking status, scores on HADS-A and AUDIT, and treatment step.

### Individual associations between serum biomarkers and prospective suicidal behaviours

In the follow-up sample (*N* = 884), increased suicidal severity and fatal/non-fatal suicide attempt during the 1-year pharmacotherapy were present in 155 (17.5%) and 38 (4.3%; 32 non-fatal, 6 fatal) participants, respectively. Baseline levels of serum biomarkers were compared by increased suicidal severity and fatal/non-fatal suicide attempt during the 12-month pharmacotherapy in Table 1. Increased suicidal severity was significantly associated with higher levels of cortisol, TNF-*α*, IL-1*β*, IL-10, and homocysteine, but with lower levels of total cholesterol, folate, and BDNF. Fatal/non-fatal suicide attempt was significantly associated with higher cortisol level, but with lower levels of total cholesterol and folate. For these biomarkers showing statistical significance, optimal cut-offs with sensitivities and specificities were obtained by AUROC analysis (Table 2). In the logistic regression analysis after adjustment for age, sex, living alone, religious affiliation, monthly income, atypical feature, number of depressive episodes, number of physical disorders, smoking status, scores on HADS-A and AUDIT, and treatment step, increased suicidal severity was independently associated with above-cut-off levels of cortisol, IL-1*β*, and homocysteine, and below-cut-off levels of total cholesterol and folate; and fatal/non-fatal suicide attempt was independently associated with above-cut-off levels of cortisol, and below-cut-off levels of total cholesterol and folate.

**Table 1.** Baseline median (interquartile range) levels of serum biomarkers by suicidal behaviour during 12-month follow-up

Serum biomarkers	Increased suicidal severity^a^		Fatal/non-fatal suicide attempt	
	Absent (*N* = 729)	Present (*N* = 155)	Absent (*N* = 846)	Present (*N* = 38)
Cortisol, μg/dL	10.5 (5.6)	**11.7 (6.1)**^†^	10.6 (5.5)	**17.7 (13.2)**^‡^
Serotonin, ng/mL	72.7 (68.2)	67.8 (62.2)	72.1 (66.6)	64.5 (108.4)
High-sensitivity C-reactive protein, mg/L	0.5 (1.0)	0.5 (1.0)	0.5 (1.0)	0.6 (0.8)
Tumor necrosis factor-*α*, pg/mL	0.6 (0.4)	0.6 (0.4)*	0.6 (0.4)	0.6 (0.4)
Interleukin-1*β*, pg/mL	1.1 (0.7)	**1.1 (0.6)**^‡^	1.1 (0.7)	0.9 (1.1)
Interleukin-6, pg/mL	1.6 (1.5)	1.7 (1.6)	1.6 (1.5)	1.7 (1.3)
Interleukin-4, pg/mL	36.9 (38.1)	36.3 (40.0)	37.0 (38.7)	35.1 (35.2)
Interleukin-10, pg/mL	10.7 (9.9)	12.3 (9.4)*	10.8 (9.8)	12.1 (12.0)
Total cholesterol, mg/dL	178.0 (49.0)	167.0 (59.0)*	177.0 (51.0)	152.0 (54.8)*
Leptin, ng/mL	5.7 (6.1)	5.3 (6.4)	5.7 (6.1)	5.0 (4.6)
Ghrelin, pg/mL	381.0 (184.5)	359.0 (177.0)	377.0 (182.9)	388.0 (159.8)
Folate, ng/mL	7.7 (6.2)	**6.3 (4.6)**^‡^	7.5 (5.9)	**4.7 (3.8)**^‡^
Homocysteine, μmol/L	10.9 (4.6)	**12.3 (4.9)**^†^	11.1 (4.7)	11.3 (5.1)
Brain derived neurotrophic factor, ng/mL	23.3 (9.0)	22.0 (7.0)*	23.0 (8.9)	21.0 (6.9)

^a^Increase in Brief Psychiatric Rating Scale suicidality item score during the follow-up compared to the baseline.

^‡^*p* < 0.001; ^†^*p* < 0.01; *p < 0.05 by using Mann–Whitney *U* tests.

**Bold style** indicates statistical significance after applying the Bonferroni correction.

**Table 2.** Serum biomarker cut-off values and probabilities of suicidal behaviour during 12-month follow-up (*N* = 884)

	Increased suicidal severity^a^				Fatal/non-fatal suicide attempt			
	Optimal cut-off	OR (95% CI)	Sensitivity (%)	Specificity (%)	Optimal cut-off	OR (95% CI)	Sensitivity (%)	Specificity (%)
Cortisol	>11.7 μg/dL	1.49 (1.03–2.14)*	51.0	60.4	>12.0 μg/dL	**7.56 (3.17**–**18.05)**^‡^	84.2	63.5
Tumor necrosis factor-*α*	>0.57 pg/mL	1.38 (0.95–2.01)	61.9	47.5	–			
Interleukin-1*β*	>0.99 pg/mL	**2.73 (1.75**–**4.26)**^‡^	83.2	39.8	–			
Interleukin-10	>9.53 pg/mL	1.50 (0.99–2.14)	64.5	44.3	–	–	–	–
Total cholesterol	<155.0 mg/dL	**2.12 (1.45**–**3.11)**^‡^	75.2	43.2	<154.0 mg/dL	**3.61 (1.74**–**7.46)**^‡^	74.6	57.9
Folate	<6.05 ng/mL	1.49 (1.01–2.18)*	67.5	57.9	<5.95 ng/mL	**2.67 (1.23**–**5.80)**^‡^	80.1	74.4
Homocysteine	>11.1 μmol/L	**1.60 (1.09**–**2.34)**^†^	60.6	53.4	–			
Brain derived neurotrophic factor	<22.8 ng/mL	1.40 (0.97–2.01)	52.7	58.1				

^a^Increase in Brief Psychiatric Rating Scale suicidality item score during the follow-up compared to the baseline.

Optimal cut-off values were obtained from the receiver operating characteristic curve.

Odds ratios (95% confidence intervals) [OR (95% CI)] were estimated by using logistic regression analyses after adjustment for age, gender, living alone, religious affiliation, monthly income, atypical feature, number of depressive episodes, number of physical disorders, smoking status, scores on Hospital Anxiety & Depression Scale-anxiety subscale and Alcohol Use Disorders Identification Test, and treatment step.

^‡^*p* < 0.001; ^†^*p* < 0.01; *p < 0.05.

**Bold style** indicates statistical significance (*p* < 0.05) after applying the Bonferroni correction.

### Multiple biomarkers and prospective suicidal behaviours

Incidences of two prospective SBs according to the increased number of biomarkers are described in the upper part of Table 3. The probability of SBs was increased incrementally with the increasing number of unfavourable biomarkers (all *p* values for trend < 0.001). Compared to the patients without any unfavourable biomarkers, the ORs (95% CIs) of those with all unfavourable biomarkers were 16.1 (2.87–90.0) and 63.3 (7.21–555.4) for increased suicidal severity and fatal/non-fatal suicide attempt, respectively in the same logistic regression model. Incidences of two prospective SBs according to the quartiles of multi-biomarker scores are described in the lower part of Table 3. The probability of SBs was increased incrementally with the higher quartile of multi-biomarker scores (all *p* values for trend < 0.001). The ORs (95% CIs) for the highest *v.* lowest quartile of multi-biomarker scores were 6.20 (3.15–12.2) and 32.3 (4.20–249.0) for increased suicidal severity and fatal/non-fatal suicide attempt, respectively in the same logistic regression model.

**Table 3.** Number of serum biomarkers, quartiles of multi-biomarker scores, and probabilities of suicidal behaviour during 12-month follow-up (*N* = 884)

	Increased suicidal severity^a^				Fatal/non-fatal suicide attempt			
	*N*	Present, *N* (%)	OR (95% CI)	*p* value for trend	*N*	Present, *N* (%)	OR (95% CI)	*p* value for trend
Number of serum biomarkers^b^								
0	60	2 (3.3)	Reference	<0.001	268	1 (0.4)	Reference	<0.001
1	214	14 (6.5)	2.10 (0.46–9.55)		381	5 (1.3)	3.30 (0.38–28.89)	
2	252	39 (15.5)	5.17 (1.20–22.26)		194	22 (11.3)	29.24 (3.76–227.47)	
3	236	59 (25.0)	9.08 (2.12–38.85)		41	10 (24.4)	63.29 (7.21–555.44)	
4	102	33 (32.4)	12.02 (2.69–53.69)					
5	20	8 (40.0)	16.06 (2.87–90.03)					
Quartiles of multi-biomarker scores^c^								
1 (lowest)	213	12 (5.6)	Reference	<0.001	268	1 (0.4)	Reference	<0.001
2	219	27 (12.3)	2.20 (1.07–4.51)		137	0 (0.0)	0.99 (0.00–0.00)	
3	245	53 (21.6)	4.29 (2.19–8.41)		244	5 (2.0)	5.36 (0.61–46.82)	
4	207	63 (30.4)	6.20 (3.15–12.19)		235	32 (13.6)	32.34 (4.20–248.99)	

a Increase in Brief Psychiatric Rating Scale suicidality item score during the follow-up compared to the baseline.

Odds ratios (95% confidence intervals) [OR (95% CI)] were estimated by using logistic regression analyses after adjustment for age, gender, living alone, religious affiliation, monthly income, atypical feature, number of depressive episodes, number of physical disorders, smoking status, scores on Hospital Anxiety & Depression Scale-anxiety subscale and Alcohol Use Disorders Identification Test, and treatment step.

b For calculating the number of serum biomarkers, 0 (favourable) or 1 (unfavourable) score from the optimal cut-offs of each significant biomarker was generated, and then summed scores were estimated ranging from 0 to 5, with higher scores indicating more unfavourable condition.

c For calculating the continuous multi-biomarker scores, the following equations were used: Increased suicidal severity = (0.331 × cortisol) + (1.108 × interleukin-1*β*) + (0.700 × total cholesterol) + (0.193 × folate) + (0.282 × homocysteine); and fatal/non-fatal suicide attempt = (1.843 × cortisol) + (1.215 × total cholesterol) + (1.010 × folate), respectively. Then, quartiles of the multi-biomarker scores were generated ranging from 1 to 4, with higher scores indicating higher risk.

### Serum biomarkers for previous and present suicidal behaviours

Individual multiple associations of serum biomarkers with previous suicidal attempt and higher baseline suicidal severity were summarised in online Supplementary Table S4~S6 using the same statistical models as for the prospective suicidal behaviours. The previous suicidal attempt was independently associated with above-cut-off levels of hsCRP and TNF-*α*, and with below-cut-off levels of total cholesterol and folate; and higher baseline suicidal severity was independently associated with above-cut-off levels of hsCRP, IL-1*β*, and IL-4, and with below-cut-off levels of folate after adjustment for age, sex, living alone, religious affiliation, monthly income, atypical feature, number of depressive episodes, number of physical disorders, smoking status, and scores on HADS-A and AUDIT (online Supplementary Table S5). The probabilities of these SBs increased incrementally with increasing numbers of unfavourable biomarkers and in higher quartiles of multi-biomarker scores (all *p* values for trend < 0.001) (online Supplementary Table S6).

## Discussion

In this study of outpatients with depressive disorders without a bipolar diagnosis, summations of multiple serum biomarker scores relating to cortisol, IL-1*β*, homocysteine, total cholesterol, and folate predicted increased suicidal severity, and summed scores relating to cortisol, total cholesterol, and folate predicted fatal/non-fatal suicide attempt during the 12-month pharmacotherapy, respectively, in a dose-dependent manner. In addition, summed scores relating to hsCRP, TNF-*α*, total cholesterol, and folate, and relating to hsCRP, IL-1*β*, IL-4, and folate, were significantly and incrementally associated with previous suicidal attempt and higher baseline suicidal severity, respectively. These associations were robust after adjustment for relevant covariates.

The role of individual blood biomarkers for explaining SBs has been unclear (Blasco-Fontecilla & Oquendo, 2016), even in meta-analyses (Black & Miller, 2015; Eisen et al., 2015; González-Castro et al., 2021; O'Connor et al., 2016; Wu et al., 2016). Similarly in this study, the individual biomarkers' sensitivities and specificities were unsatisfactory for clinical application, even though predictive and discriminant values for SBs estimated by ORs (95% CIs) were statistically significant (Table 2 and online Supplementary Table S5). A particular observation of this study was that biomarkers in combination had significantly better and incremental predictive values for prospectively ascertained SBs. The ORs of patients with all unfavourable biomarkers was 16.1 and 63.3 compared to those without, and those of the lowest *v.* highest quartile of multi-biomarker scores were 6.2 and 32.3 for increased suicidal severity and fatal/non-fatal suicide attempt, respectively (Table 3). These were remarkable improvements for the ORs of individual markers shown 1.5~2.7 for increased suicidal severity and shown 2.7~7.6 for fatal/non-fatal suicide attempt. The same was found with previous and current SBs (online Supplementary Table S6).

Many researchers have argued the importance and necessity of multiple biomarkers for SB risk assessment (Sudol & Mann, 2017). In this respect, ‘omics’ approaches might provide a solution; however, these studies have reported mixed findings (Le-Niculescu et al., 2013; Mullins et al., 2014; Perroud et al., 2012). Some investigators have examined combinations of two biological risk factors simultaneously: for example, a coupling of cortisol- and serotonin or cholesterol- related factors (Coryell & Schlesser, 2007; Jokinen et al., 2008, 2009; Mann et al., 2006). However, the explanatory power of the combinations for SBs has still been unsatisfactory (Blasco-Fontecilla & Oquendo, 2016). The role of multiple peripheral blood biomarkers has rarely been evaluated and this approach is thus not fully understood. An exception lies in studies evaluating varieties of cytokines and chemokines (Isung, Mobarrez, Nordström, Asberg, & Jokinen, 2012; Janelidze et al., 2013); however, these did not evaluate combined effects. As far as we aware, the present study is the first to investigate the potential multi-modal effects of blood biomarkers covering various functional systems on SBs.

Almost all previous studies in this kind have also been limited by cross-sectional case–control designs, comparing blood biomarker levels by histories of suicidal attempts or by the severity of current suicidality (O'Connor et al., 2016; Wu et al., 2016). Some studies have evaluated prospective increases in suicidal ideation during short term 8~12-week antidepressant treatment (Perroud et al., 2012) and, as far as we aware, ours is the first study to investigate prospective associations of blood biomarker levels with fatal/non-fatal suicidal attempts as well as increased suicidal severity over a relatively long-term 12-month treatment period. Three serum biomarkers – cortisol, total cholesterol, and folate – were identified as significant predictors of both prospective SBs. Cortisol has been extensively investigated as a SB biomarker for stress, but two recent meta-analyses reported overall no associations between cortisol levels and suicidal attempts due to the controversial findings among the studies (Hernandez-Diaz et al., 2020; O'Connor et al., 2016). Our finding of a lack of associations of cortisol levels with previous suicide attempts after adjustment is in agreement with these meta-analyses. From these findings, serum cortisol levels might be a predictive rather than a retrospective biomarker of SB in depressive patients receiving pharmacotherapy. Total cholesterol has also been investigated repeatedly based on the cholesterol-serotonin hypothesis. A meta-analysis identified an inverse association between serum total cholesterol levels and suicidality (Wu et al., 2016), consistent with our findings on the independent association with previous suicidal attempt. Folate is involved in methylation reactions necessary for the production of monoamine neurotransmitters, phospholipids, and nucleotides, Folate deficiency has been associated with depressive disorders (Kim et al., 2008) and folate intake has been associated with augmentation of antidepressant effects (Sarris et al., 2016). Folate thus might plausibly be associated with SB, although this has received relatively little evaluation to date. The present findings suggest that serum total cholesterol and folate levels are significantly associated with prospective as well as previous SBs. However, the novel findings on prospective SBs need further replication.

Additionally, serum IL-1*β* and homocysteine levels were significantly associated with increased suicidal severity. A meta-analysis of cytokines and chemokines in suicidality reported that levels of IL-1*β* and IL-6 were significantly increased in blood samples of patients with suicidality compared with both patients without suicidality and healthy controls (Black & Miller, 2015), consistent with our findings for baseline SB. In addition, our findings indicate that serum IL-1*β* levels might also be used as a predictive marker of prospective SB. Homocysteine, also involved in methylation reactions as with folate, has been associated with depressive disorders (Kim et al., 2008), but has not been evaluated as a biomarker for suicidality. Our significant findings on increased suicidal severity should be considered as empirical, since the statistical significance was found just one of four SBs and it was novel.

Previous suicidal attempt and higher baseline suicidal severity were independently associated hsCRP, TNF-*α*, total cholesterol, and folate, and hsCRP, IL-1*β*, IL-4, and folate, respectively. Other than biomarkers also predicting both two SBs (total cholesterol, and folate), all biomarkers associated with previous and present SBs were included in immune and inflammatory systems (hsCRP, IL-1*β*, and IL-4). As stated above, this kind of cytokine biomarkers were found to be significantly associated with previous and contemporary SBs in a meta-analysis (Black & Miller, 2015), assumed to be due to the effects of cytokines on kynurenine pathway of tryptophan degradation or on glutaminergic neurotransmission through tryptophan catabolism (Serafini et al., 2013). In addition, cytokine imbalance hypotheses are widely accepted in depressive disorders (Dowlati et al., 2010). Since all participants in this study were patients with depressive disorders, cytokine markers might be overrepresented particularly for previous and contemporary SBs.

There are several limitations in this study. First, since the study design was naturalistic, depression treatment was decided by patient preference with a physician's guidance, rather than using an imposed pre-established protocol. Thus, our results can only provide broad and general biomarkers for predicting SBs outcomes within the context of a variety of pharmacotherapy regimes. Second, biomarkers evaluated were frequently investigated and relatively well-known ones (Sudol & Mann, 2017) rather than a novel, so there is scope for further investigation. Third, the biomarkers were only examined at baseline and no attempt was made to account for changes in levels of some biomarkers according to treatment responses (Martinotti et al., 2016; Yoshimura et al., 2009). However, in this respect, the present study focused on predictive rather than reflective values of biomarkers for SBs. Fourth, the numerical differences of biomarkers between participants with and without SBs were very small, although they were statistically significant. Fifth, there was considerable sample attrition during the 12-month treatment period. Because of poor prognostic characteristics among participants who were lost to follow-up, such as unemployed status and melancholic features, these participants presumably would have attenuated (rather than exaggerated) the observed findings. Sixth, recruitment was carried out at a single site, which may limit the generalisability of the findings, although a single centre study has potential strengths in terms of consistency in evaluation and treatment. Seventh, the number of fatal/non-fatal suicide events during the 12-month pharmacotherapy was too small to analyse separately, and therefore more long-term follow-up is needed. Eighth, the prospective SB outcomes of the present study didn't consider previous reports on possible increases in SB s related to antidepressant treatment particularly at the early stage of (Perlis et al., 2007). Ninth, the age range was broad (17~85) despite of the previous reports on the age-specific differences in the associations with biomarker levels, SBs, and pharmacotherapeutic responses (Calati, Nemeroff, Lopez-Castroman, Cohen, & Galynker, 2020).

This study had multiple strengths, including its novel combined retrospective and prospective design for the evaluation of SBs. The sample size was large compared to previous biomarker studies, and participants were evaluated with a structured research protocol and well-recognised and standardised scales. As stated above, this study is the first, to our knowledge, to report on predictive values of biomarkers in combination covering several functional systems. In addition, diverse covariates were considered, which we hope will have improved the robustness of the study findings.

## Conclusion

Identification of individuals at risk of suicide is mostly based upon subjective data so far. The introduction of biomarkers could add objectivity to the prediction of SBs. This study suggests that combinations of serum biomarkers relating to cortisol, total cholesterol, and folate, as well as IL-1*β* and homocysteine could considerably improve the predictability of fatal/non-fatal suicide attempt and increased suicidal severity, respectively. These findings could be translated in clinical practice for treating outpatients with depressive disorders, since they were drawn from a naturalistic, prospective design, maximising resemblance to real-world clinical situations. It is reasonable to recommend that patients with unfavourable biomarkers are monitored frequently and treated carefully to prevent SBs. The observed suicide rate during the 1-year follow-up was 678 per 100 000 (6/884), approximately 27 times higher than that (24.6 per 100 000) of all South Korea in 2019 (OECD, 2021). Our findings may have clinical utility in screening and identifying depression associated with high suicidality. Given the multiple determinants of suicide, a combination of blood-based, neuropsychological, and neuroimaging factors might yield a better estimate of risk. The novel findings presented here on serum biomarkers specifically could be considered as a component of future comprehensive studies and prevention guidelines.

## Supplementary material

For supplementary material accompanying this paper visit https://doi.org/10.1017/S0033291722001180. click here to view supplementary material
