# Supplementary material for: Multiple serum biomarkers for predicting suicidal behaviours in depressive patients receiving pharmacotherapy
Source: Psychol Med. 2022 May 17;53(10):4385–94. doi: 10.1017/S0033291722001180 (PMC10388309; doi:10.1017/S0033291722001180)
Supplement: Supplementary file 1 [file S0033291722001180sup001.docx]

Multiple serum biomarkers for predicting suicidal behaviours in depressive patients receiving pharmacotherapy

Jae-Min Kim^*^, Hee-Ju Kang^*^, Ju-Wan Kim, Wonsuk Choi, Ju-Yeon Lee, Sung-Wan Kim, Il-Seon Shin, Min-Gon Kim, Byung Jo Chun, Robert Stewart. Multiple serum biomarkers for predicting suicidal behaviours in depressive patients receiving pharmacotherapy

**Supplementary Information**

**Table S1…….……………....………………………………………………………………..2**

**Table S2………………………….……………....…………………………………………..3**

**Table S3………………………….……………....…………………………………………..4**

**Table S4………………………….……………....…………………………………………..5**

**Table S5………………………….……………....…………………………………………..6**

**Table S6………………………….……………....…………………………………………..7**

| **Table S1** Descriptive characteristics of the baseline and followed up samples | | |
| --- | --- | --- |
|  | **Baseline sample (N=1094)** | **Followed up sample (N=884)** |
| **Socio-demographic characteristics** |  |  |
| Age, mean (SD) / range years | 56.9 (14.9) / 17~85 | 57.0 (14.8) / 17~85 |
| Gender, N (%) female | 753 (68.8) | 607 (68.7) |
| Education, mean (SD) / range years | 9.1 (4.8) / 0~20 | 9.1 (4.8) / 0~20 |
| Marital status, N (%) unmarried | 327 (29.9) | 257 (29.1) |
| Living alone, N (%) | 167 (15.3) | 131 (14.8) |
| Religious non-affiliation, N (%) | 483 (44.1) | 388 (43.9) |
| Unemployed status, N (%) | 316 (28.9) | 242 (27.4) |
| Monthly income, N (%) <2,000 USD | 653 (59.7) | 522 (59.0) |
| **Clinical characteristics** |  |  |
| Major depressive disorder, N (%) | 933 (85.3) | 761 (86.1) |
| Melancholic feature, N (%) | 165 (15.1) | 141 (16.0) |
| Atypical feature, N (%) | 69 (6.3) | 55 (6.2) |
| Age at onset, mean (SD) / range years | 50.9 (16.6) / 8~85 | 50.8 (16.4) / 8~85 |
| Duration of illness, mean (SD) / range years | 6.3 (9.3) / 0~57 | 6.4 (9.4) / 0~57 |
| Number of depressive episodes, mean (SD) / range | 2.3 (5.0) / 0~40 | 2.3 (5.0) / 0~40 |
| Duration of present episode, mean (SD) / range months | 7.4 (10.4) / 0~90 | 7.6 (10.4) / 0~90 |
| Family history of depression, N (%) | 160 (14.6) | 125 (14.1) |
| Number of physical disorders, mean (SD) / range | 1.6 (1.3) / 0~5 | 1.7 (1.3) / 0~5 |
| Current smoker, N (%) | 45 (4.1) | 37 (4.2) |
| **Assessment scales,** mean (SD) |  |  |
| Hospital Anxiety & Depression Scale-  depression subscale | 13.7 (3.9) / 2~21 | 13.7 (3.9) / 2~21 |
| Hospital Anxiety & Depression Scale-  anxiety subscale | 11.8 (4.0) / 1~21 | 11.8 (4.0) / 1~21 |
| Alcohol Use Disorders Identification Test | 5.4 (8.9) / 1~46 | 5.4 (9.1) / 1~46 |
| **Treatment step over 12-month**^a^ (N=884), N (%) | |  |
| Step 1 |  | 326 (36.9) |
| Step 2 |  | 286 (32.4) |
| Step 3 |  | 172 (19.5) |
| Step 4 |  | 100 (11.3) |
| ^a^For patients with an insufficient response or uncomfortable side effects, next treatment steps (1, 2, 3, and 4 or over) with alternative strategies (switching, augmentation, combination, and mixtures of these approaches) were administered considering measurements and patient preference at 3, 6, 9, and 12 weeks, and at 6, 9, and 12 months. | | |

| **Table S2** Characteristics compared by previous suicidal attempt before the study enrolment. | | | | |
| --- | --- | --- | --- | --- |
|  | **Absent**  **(N=998)** | **Present**  **(N=96)** | **Statistical**  **coefficients** | **P-value** |
| **Socio-demographic characteristics** |  |  |  |  |
| Age, mean (SD) years | 57.9 (14.4) | 46.9 (15.5) | t=+7.124 | **<0.001** |
| Gender, N (%) male | 301 (30.2) | 40 (41.7) | χ^2^=5.404 | **0.020** |
| Education, mean (SD) years | 8.9 (4.8) | 10.9 (4.5) | t=-3.857 | **<0.001** |
| Marital status, N (%) unmarried | 285 (28.6) | 42 (43.8) | χ^2^=9.646 | **0.002** |
| Living alone, N (%) | 150 (15.0) | 17 (17.7) | χ^2^=0.486 | 0.486 |
| Religious non-affiliation, N (%) | 422 (42.3) | 61 (63.5) | χ^2^=16.049 | **<0.001** |
| Unemployed status, N (%) | 288 (28.9) | 28 (29.2) | χ^2^=0.004 | 0.949 |
| Monthly income, N (%) <2,000 USD | 609 (61.0) | 44 (45.8) | χ^2^=8.397 | **0.004** |
| **Clinical characteristics** |  |  |  |  |
| Major depressive disorder, N (%) | 843 (84.5) | 90 (93.8) | χ^2^=6.010 | **0.014** |
| Melancholic feature, N (%) | 156 (15.6) | 9 (9.4) | χ^2^=2.676 | 0.102 |
| Atypical feature, N (%) | 53 (5.3) | 16 (16.7) | χ^2^=19.112 | **<0.001** |
| Age at onset, mean (SD) years | 53.2(16.1) | 38.0 (16.5) | t=+8.810 | **<0.001** |
| Duration of illness, mean (SD) years | 4.7 (8.7) | 8.8 (11.6) | t=-3.415 | **0.001** |
| Number of depressive episodes, mean (SD) | 1.0 (1.4) | 2.1 (2.0) | t=-5.426 | **<0.001** |
| Duration of present episode, mean (SD) months | 7.2 (10.4) | 9.1 (9.7) | t=-1.682 | 0.093 |
| Family history of depression, N (%) | 146 (14.6) | 14 (14.6) | χ^2^=0.001 | 0.990 |
| Number of physical disorders, mean (SD) | 1.7 (1.3) | 1.3 (1.3) | t=+2.453 | 0.114 |
| Current smoker, N (%) | 102 (10.2) | 21 (21.9) | χ^2^=15.037 | **0.001** |
| **Assessment scales**, mean (SD) scores |  |  |  |  |
| Hospital Anxiety & Depression Scale-depression subscale | 13.6 (3.9) | 14.3 (4.2) | t=-1.777 | 0.076 |
| Hospital Anxiety & Depression Scale-anxiety subscale | 11.7 (4.0) | 12.8 (4.3) | t=-2.492 | **0.013** |
| Alcohol Use Disorders Identification Test | 4.7 (8.3) | 11.7 (12.0) | t=-5.576 | **<0.001** |
| **Treatment step over 12-month**^a^ (N=884), N (%) | |  |  |  |
| Step 1 | 303 (37.3) | 23 (31.9) | χ^2^=10.151 | **0.017** |
| Step 2 | 268 (33.0) | 18 (25.0) |  |  |
| Step 3 | 157 (19.3) | 15 (20.3) |  |  |
| Step 4 | 84 (10.3) | 16 (22.2) |  |  |

^a^For patients with an insufficient response or uncomfortable side effects, next treatment steps (1, 2, 3, and 4 or over) with alternative strategies (switching, augmentation, combination, and mixtures of these approaches) were administered considering measurements and patient preference at 3, 6, 9, and 12 weeks, and at 6, 9, and 12 months.

| **Table S3** Characteristics compared by Brief Psychiatric Rating Scale (BPRS) suicidality item score at baseline | | | | |
| --- | --- | --- | --- | --- |
|  | **0~2**  **(N=732)** | **3~7**  **(N=362)** | **Statistical coefficients** | **P-value** |
| **Socio-demographic characteristics** |  |  |  |  |
| Age, mean (SD) years | 58.1 (14.0) | 54.6 (16.2) | t=+3.508 | **<0.001** |
| Gender, N (%) female | 503 (68.7) | 250 (69.1) | χ^2^=0.013 | 0.908 |
| Education, mean (SD) years | 9.1 (4.7) | 9.0 (4.9) | t=+0.287 | 0.774 |
| Marital status, N (%) unmarried | 198 (27.0) | 129 (35.6) | χ^2^=8.521 | **0.004** |
| Living alone, N (%) | 100 (13.7) | 67 (18.5) | χ^2^=4.399 | **0.036** |
| Religious non-affiliation, N (%) | 297 (40.6) | 186 (51.4) | χ^2^=11.473 | **0.001** |
| Unemployed status, N (%) | 195 (26.6) | 121 (33.4) | χ^2^=5.430 | **0.020** |
| Monthly income, N (%) <2,000 USD | 427 (58.3) | 226 (62.4) | χ^2^=1.690 | 0.194 |
| **Clinical characteristics** |  |  |  |  |
| Major depressive disorder, N (%) | 606 (82.8) | 327 (90.3) | χ^2^=10.985 | **0.001** |
| Melancholic feature, N (%) | 109 (14.9) | 56 (15.5) | χ^2^=0.063 | 0.801 |
| Atypical feature, N (%) | 38 (5.2) | 31 (8.6) | χ^2^=4.661 | **0.031** |
| Age at onset, mean (SD) years | 53.5 (16.2) | 48.6 (17.3) | t=+4.627 | **<0.001** |
| Duration of illness, mean (SD) years | 4.6 (8.6) | 6.0 (9.9) | t=-2.341 | **0.020** |
| Number of depressive episodes, mean (SD) | 1.0 (1.4) | 1.3 (1.5) | t=-3.868 | **<0.001** |
| Duration of present episode, mean (SD) months | 7.1 (9.7) | 7.9 (11.6) | t=-1.179 | 0.239 |
| Family history of depression, N (%) | 113 (15.4) | 47 (13.0) | χ^2^=1.168 | 0.280 |
| Number of physical disorders, mean (SD) | 1.6 (1.2) | 1.7 (1.3) | t=-0.522 | 0.602 |
| Current smoker, N (%) | 64 (8.7) | 59 (16.3) | χ^2^=8.914 | **0.003** |
| **Assessment scales**, mean (SD) scores |  |  |  |  |
| Hospital Anxiety & Depression Scale-depression subscale | 12.9 (4.0) | 15.2 (3.4) | t=-9.961 | **<0.001** |
| Hospital Anxiety & Depression Scale-anxiety subscale | 10.9 (3.9) | 13.6 (3.7) | t=-11.123 | **<0.001** |
| Alcohol Use Disorders Identification Test | 5.1 (8.6) | 5.8 (9.3) | t=-1.183 | 0.237 |
| **Treatment step over 12-month**^a^ (N=884), N (%) | |  |  |  |
| Step 1 | 233 (39.2) | 93 (32.1) | χ^2^=7.271 | **0.007** |
| Step 2 | 193 (32.5) | 93 (32.1) | (linear) |  |
| Step 3 | 110 (18.5) | 62 (21.4) |  |  |
| Step 4 | 58 (9.8) | 42 (14.5) |  |  |
| ^a^For patients with an insufficient response or uncomfortable side effects, next treatment steps (1, 2, 3, and 4 or over) with alternative strategies (switching, augmentation, combination, and mixtures of these approaches) were administered considering measurements and patient preference at 3, 6, 9, and 12 weeks, and at 6, 9, and 12 months. | | | | |

| **Table S4** Baseline median (interquartile range) levels of serum biomarkers by suicidal behavior evaluated at baseline. | | | | | |
| --- | --- | --- | --- | --- | --- |
| **Serum biomarkers** | **Previous suicidal attempt** | |  | **Higher baseline suicidal severity^a^** | |
|  | **Absent (N=998)** | **Present (N=96)** |  | **Absent (N=732)** | **Present (N=362)** |
| Cortisol, μg/dL | 10.6 (5.7) | 11.1 (6.6)^*^ |  | 10.4 (5.6) | 11.1 (6.5)^*^ |
| Serotonin, ng/mL | 73.8 (66.1) | 66.9 (84.5) |  | 74.5 (64.6) | 67.4 (77.9) |
| High-sensitivity C-reactive protein, mg/L | 0.4 (1.0) | 0.6 (1.2)^*^ |  | 0.4 (1.0) | **0.6 (1.2)**^‡^ |
| Tumor necrosis factor-α, pg/mL | 0.6 (0.4) | 0.7 (0.5)^*^ |  | 0.6 (0.4) | 0.6 (0.5) |
| Interleukin-1β, pg/mL | 1.1 (0.7) | **1.3 (0.9)**^†^ |  | 1.1 (0.7) | 1.2 (0.8)^†^ |
| Interleukin-6, pg/mL | 1.6 (1.5) | 1.8 (2.2) |  | 1.6 (1.6) | 1.7 (1.7) |
| Interleukin-4, pg/mL | 37.2 (38.2) | 35.3 (35.2) |  | 35.0 (34.1) | **42.9 (42.3)**^‡^ |
| Interleukin-10, pg/mL | 10.7 (9.7) | 11.0 (10.8) |  | 10.3 (9.9) | 11.0 (9.6) |
| Total cholesterol, mg/dL | 178.0 (51.3) | 168.0 (63.5)^*^ |  | 177.0 (53.8) | 178.0 (52.0) |
| Leptin, ng/mL | 5.8 (6.2) | 5.6 (5.7) |  | 6.0 (6.2) | 6.0 (6.2) |
| Ghrelin, pg/mL | 377.0 (178.8) | 404.5 (197.3) |  | 384.5 (188.5) | 371.5 (165.3) |
| Folate, ng/mL | 7.6 (6.0) | **5.6 (5.0)**^‡^ |  | 7.8 (6.1) | **6.8 (5.5)**^‡^ |
| Homocysteine, μmol/L | 10.9 (4.8) | 10.9 (3.8) |  | 11.0 (4.7) | 10.8 (4.7) |
| Brain derived neurotrophic factor, ng/mL | 23.5 (8.6) | 22.0 (8.4) |  | 23.7 (8.8) | 22.7 (8.5) |
| ^a^Brief Psychiatric Rating Scale suicidality item score 4 (moderate) ~ 7 (extremely severe).  ^‡^P<0.001; ^†^P<0.01; ^*^P<0.05 by using Mann-Whitney U tests.  **Bold style** indicates statistical significance after applying the Bonferroni correction. | | | | | |

| **Table S5** Serum biomarker cut-off values and probabilities of suicidal behavior at baseline (N=1094). | | | | | | | | | |
| --- | --- | --- | --- | --- | --- | --- | --- | --- | --- |
|  | **Previous suicidal attempt** | | | |  | **Higher baseline suicidal severity^a^** | | | |
|  | **Optimal cut-off** | **OR (95% CI)** | **Sensitivity** | **Specificity** |  | **Optimal cut-off** | **OR (95% CI)** | **Sensitivity** | **Specificity** |
| Cortisol | >12.1μg/dL | 1.18 (0.74-1.88) | 47.9% | 64.4% |  | >10.1μg/dL | 1.18 (0.89-1.56) | 59.9% | 47.7% |
| High-sensitivity C-reactive protein | >0.48mg/dL | 1.70 (1.06-2.72)^†^ | 60.4% | 53.7% |  | >0.60mg/dL | **1.85 (1.39-2.45)**^†^ | 50.6% | 65.3% |
| Tumor necrosis factor-α | >0.55pg/mL | **1.98 (1.21-3.22)**^†^ | 67.7% | 45.0% |  | - |  |  |  |
| Interleukin-1β | >1.20pg/mL | 1.30 (0.81-2.08) | 59.4% | 59.7% |  | >0.93pg/mL | 1.43 (1.05-1.95)^*^ | 78.5% | 33.6% |
| Interleukin-4 | - |  |  |  |  | >28.7pg/mL | 1.53 (1.14-2.06)^*^ | 70.4% | 39.8% |
| Total cholesterol | <144.0mg/dL | **2.64 (1.59-4.37)**^‡^ | 82.4% | 35.4% |  | - | - | - | - |
| Folate | <6.15ng/mL | **1.89 (1.17-3.07)**^†^ | 70.2% | 63.3% |  | <6.25ng/mL | 1.53 (1.15-2.05)^*^ | 65.4% | 59.4% |
| ^a^Brief Psychiatric Rating Scale suicidality item score 4 (moderate) ~ 7 (extremely severe).  Optimal cut-off values were obtained from the receiver operating characteristic curve.  Odds ratios (95% confidence intervals) [OR (95% CI)] were estimated by using logistic regression analyses after adjustment for age, gender, living alone, religious affiliation, monthly income, atypical feature, number of depressive episodes, number of physical disorders, smoking status, and scores on Hospital Anxiety & Depression Scale-anxiety subscale and Alcohol Use Disorders Identification Test.  ^‡^P<0.001; ^†^P<0.01; ^*^P<0.05.  **Bold style** indicates statistical significance (P<0.05) after applying the Bonferroni correction. | | | | | | | | | |

| **Table S6** Number of serum biomarkers, quartiles of multi-biomarker scores, and probabilities of suicidal behavior at baseline (N=1094). | | | | | | | | | |
| --- | --- | --- | --- | --- | --- | --- | --- | --- | --- |
|  | **Previous suicidal attempt** | | | |  | **Higher baseline suicidal severity^a^** | | | |
|  | **N** | **Present,**  **N (%)** | **OR (95% CI)** | **P-value for trend** |  | **N** | **Present,**  **N (%)** | **OR (95% CI)** | **P-value for trend** |
| **Number of serum biomarkers^b^** |  |  |  |  |  |  |  |  |  |
| 0 | 191 | 5 (2.6) | Reference | <0.001 |  | 74 | 14 (18.9) | Reference | <0.001 |
| 1 | 328 | 21 (6.4) | 2.48 (0.89-6.94) |  |  | 240 | 56 (23.3) | 1.19 (0.60-2.37) |  |
| 2 | 326 | 27 (8.3) | 2.90 (1.05-7.98) |  |  | 372 | 114 (30.6) | 1.86 (0.96-3.59) |  |
| 3 | 214 | 34 (15.9) | 7.09 (2.54-19.76) |  |  | 299 | 116 (38.8) | 2.35 (1.20-4.59) |  |
| 4 | 35 | 9 (25.7) | 13.83 (3.85-49.73) |  |  | 109 | 62 (56.9) | 4.84 (2.29-10.25) |  |
| **Quartiles of multi-biomarker scores^c^** |  |  |  |  |  |  |  |  |  |
| 1 (lowest) | 276 | 10 (3.6) | Reference | <0.001 |  | 295 | 66 (22.4) | Reference | <0.001 |
| 2 | 208 | 12 (5.8) | 1.73 (0.71-4.22) |  |  | 275 | 80 (29.1) | 1.43 (0.96-2.14) |  |
| 3 | 346 | 26 (7.5) | 1.99 (0.91-4.36) |  |  | 188 | 62 (33.0) | 1.75 (1.12-2.73) |  |
| 4 | 264 | 48 (18.2) | 5.84 (2.71-12.50) |  |  | 336 | 154 (45.8) | 2.74 (1.87-4.00) |  |
| ^a^Brief Psychiatric Rating Scale suicidality item score 4 (moderate) ~ 7 (extremely severe).  Odds ratios (95% confidence intervals) [OR (95% CI)] were estimated by using logistic regression analyses after adjustment for age, gender, living alone, religious affiliation, monthly income, atypical feature, number of depressive episodes, number of physical disorders, smoking status, and scores on Hospital Anxiety & Depression Scale-anxiety subscale and Alcohol Use Disorders Identification Test.  ^b^For calculating the number of serum biomarkers, 0 (favourable) or 1 (unfavourable) score from the optimal cut-offs of each significant biomarker was generated, and then summed scores were estimated ranging from 0 to 5, with higher scores indicating more unfavourable condition..  ^c^For calculating the continuous multi-biomarker scores, the following equations were used: Previous suicide attempt = (0.422 x high-sensitivity C-reactive protein) + (0.544 x tumor necrosis factor-α) + (0.978 x total cholesterol) + (0.584x folate); and higher baseline suicidal severity= (0.519 x high-sensitivity C-reactive protein) + (0.128x interleukin-1β) + (0.446 x interleukin-4) + (0.458x folate), respectively. Then, quartiles of the multi-biomarker scores were generated ranging from 1 to 4, with higher scores indicating higher risk. | | | | | | | | | |
